# Ultrasensitive CRISPR-based diagnostic for field-applicable detection of *Plasmodium* species in symptomatic and asymptomatic malaria

**DOI:** 10.1073/pnas.2010196117

**Published:** 2020-09-21

**Authors:** Rose A. Lee, Helena De Puig, Peter Q. Nguyen, Nicolaas M. Angenent-Mari, Nina M. Donghia, James P. McGee, Jeffrey D. Dvorin, Catherine M. Klapperich, Nira R. Pollock, James J. Collins

**Affiliations:** ^a^Wyss Institute for Biologically Inspired Engineering, Harvard University, Boston, MA 02115;; ^b^Division of Infectious Diseases, Department of Pediatrics, Boston Children’s Hospital, Boston, MA 02115;; ^c^Division of Infectious Diseases, Department of Medicine, Beth Israel Deaconess Medical Center, Boston, MA 02215;; ^d^Institute for Medical Engineering and Science, Department of Biological Engineering, Massachusetts Institute of Technology, Cambridge, MA 02139;; ^e^School of Engineering and Applied Sciences, Harvard University, Cambridge, MA 02138;; ^f^Department of Biomedical Engineering, Boston University, Boston, MA 02215;; ^g^Department of Laboratory Medicine, Boston Children’s Hospital, Boston, MA 02115;; ^h^Infectious Disease and Microbiome Program, Broad Institute of MIT and Harvard, Cambridge, MA 02142

**Keywords:** CRISPR-Dx, diagnostics, malaria, SHERLOCK

## Abstract

Asymptomatic carriers of *Plasmodium* parasites hamper malaria control and eradication. Achieving malaria eradication requires ultrasensitive diagnostics for low parasite density infections (<100 parasites per microliter blood) that work in resource-limited settings (RLS). Sensitive point-of-care diagnostics are also lacking for nonfalciparum malaria, which is characterized by lower density infections and may require additional therapy for radical cure. Molecular methods, such as PCR, have high sensitivity and specificity, but remain high-complexity technologies impractical for RLS. Here we describe a CRISPR-based diagnostic for ultrasensitive detection and differentiation of *Plasmodium falciparum*, *Plasmodium vivax*, *Plasmodium ovale*, and *Plasmodium malariae*, using the nucleic acid detection platform SHERLOCK (specific high-sensitivity enzymatic reporter unlocking). We present a streamlined, field-applicable, diagnostic comprised of a 10-min SHERLOCK parasite rapid extraction protocol, followed by SHERLOCK for 60 min for *Plasmodium* species-specific detection via fluorescent or lateral flow strip readout. We optimized one-pot, lyophilized, isothermal assays with a simplified sample preparation method independent of nucleic acid extraction, and showed that these assays are capable of detection below two parasites per microliter blood, a limit of detection suggested by the World Health Organization. Our *P. falciparum* and *P. vivax* assays exhibited 100% sensitivity and specificity on clinical samples (5 *P. falciparum* and 10 *P. vivax* samples). This work establishes a field-applicable diagnostic for ultrasensitive detection of asymptomatic carriers as well as a rapid point-of-care clinical diagnostic for nonfalciparum malaria species and low parasite density *P. falciparum* infections.

## Significance

Detection of submicroscopic malaria in asymptomatic individuals is needed for eradication and remains a diagnostic gap in resource-limited settings. Nonfalciparum clinical diagnostics are a second gap, as these infections have a low parasite density and are commonly undetected. We describe an integrated, 60-min, ultrasensitive and specific CRISPR-based diagnostic for the four major pathogenic *Plasmodium* species that can fill these gaps. Using the SHERLOCK (specific high-sensitivity enzymatic reporter unlocking) platform, we designed assays with limits of detection below that recommended by the World Health Organization. These assays have a simplified sample preparation method: the SHERLOCK parasite rapid extraction protocol, which eliminates complicated nucleic acid extraction steps. Our work further translates the SHERLOCK platform into a field-deployable diagnostic.

Malaria has an enormous global health impact, with an estimated 228 million cases and 405,000 deaths in 2018 (1). In 2007, the World Health Organization (WHO) endorsed the ambitious goal of eradicating malaria, but the decline has stalled and even reversed in some regions since 2014 (1). Malaria control strategies are thwarted in part by asymptomatic carriers, who serve as parasite reservoirs for ongoing spread. Low-density infections (<100 parasites per microliter blood) are particularly common in low-endemnicity settings, and fall below the limit of detection (LOD) of both light microscopy and antigen-based malaria rapid diagnostic tests (RDT), which are the primary diagnostics used worldwide. Submicroscopic carriers may be responsible for 20 to 50% of all human-to-mosquito transmission (2).

Highly sensitive, field-applicable diagnostic devices compatible for use in resource-limited settings (RLS) are required for detection of residual infections in preelimination areas. The Malaria Eradication Research Agenda Consultative Group on Diagnoses and Diagnostics and the WHO determined that a diagnostic capable of detecting two parasites per microliter would be a “significant improvement on expert microscopy” (3, 4).

While light microscopy remains the gold standard for distinguishing *Plasmodium* species, it requires skilled technician interpretation and is time-intensive. RDT strengths include point-of-care (POC) utility and an intuitive format, but most tests target *Plasmodium falciparum* and are incapable of species-specific identification, a critical clinical limitation as *Plasmodium vivax* and *Plasmodium ovale* uniquely require an 8-aminoquinolone (i.e., primaquine or tafenoquine) therapy to prevent relapse. Although there are sustained calls for more sensitive, nonfalciparum malaria diagnostics, this remains an ongoing diagnostic gap (5). Additionally, the most common RDT antigen target for *P. falciparum*, histidine-rich protein 2 (HRP2), persists for several weeks after resolution of infection, contributing to false-positives and limited surveillance utility (6). A worrisome rise in *hrp2* gene deletions over the past two decades also renders many RDTs obsolete (40% of parasites in some areas of South America) (7, 8).

Molecular methods for DNA detection, such as PCR, are capable of much higher sensitivity and specificity, confirmed by surveillance surveys where the prevalence of infection estimated by light microscopy was half of that measured by PCR (9). Yet PCR remains a high-complexity technology requiring expensive laboratory equipment, personnel training, and nucleic acid extraction sample preparation, making it impractical for RLS. The furthest developed commercial nucleic acid amplification tests (NAAT) for malaria are loop-mediated isothermal amplification-based assays, but they have exhibited disappointing sensitivity in field studies in comparison to PCR and require separate nucleic acid extraction steps (10–12).

Most NAATs for pathogen detection require nucleic acid extraction via multistep commercial kits involving numerous specimen transfers, laboratory infrastructure (flow-columns, management of biohazardous wastes such as chaotropic agents, and so forth), and 30 min or more of preassay preparation time. This is not practically implementable for POC testing, and sample preparation remains a general bottleneck for adoption of nucleic acid technologies, particularly for RLS (13, 14).

Here, we describe the development of field-applicable, 60-min, ultrasensitive malaria diagnostic tools using the CRISPR-based nucleic acid detection platform SHERLOCK (specific high-sensitivity enzymatic reporter unlocking) (15–19) for detection of *P. falciparum*, *P. vivax*, *P. ovale*, and *Plasmodium malariae*. Our isothermal, lyophilized, one-pot SHERLOCK assays for ultrasensitive detection are coupled with a simplified sample preparation method: S-PREP (SHERLOCK parasite rapid extraction protocol) that eliminates the need for commercial kit nucleic acid extraction. Building from prior work on a *P. falciparum* SHERLOCK assay, we demonstrate a simplified field-ready SHERLOCK diagnostic, and confirm the accuracy of our diagnostic on simulated whole blood, serum, and dried blood spot (DBS) samples, as well as clinical samples from patients with *P. falciparum* and *P. vivax* infection.

## Results

### Design and Optimization of Malaria SHERLOCK Diagnostic.

**Fig. 1.**
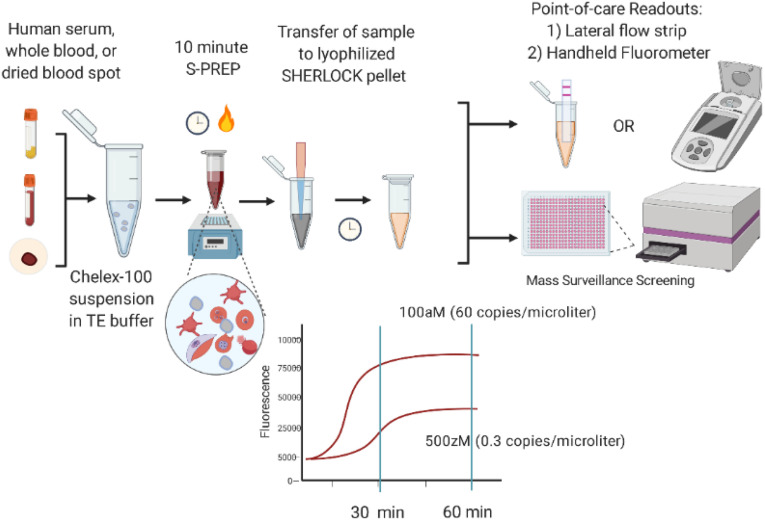
SHERLOCK diagnostic workflow: 1) Human serum, whole blood, or DBS samples undergo a 10-min S-PREP protocol where the sample is suspended in 20% (wt/vol) Chelex-100 in TE buffer with 50 mM DTT and incubated at 95 °C for 10 min; and 2) transfer of suspended sample to lyophilized SHERLOCK pellet followed by incubation at 40 °C for 60 min prior to endpoint analysis via fluorescence or lateral flow strip.

**Fig. 2.**
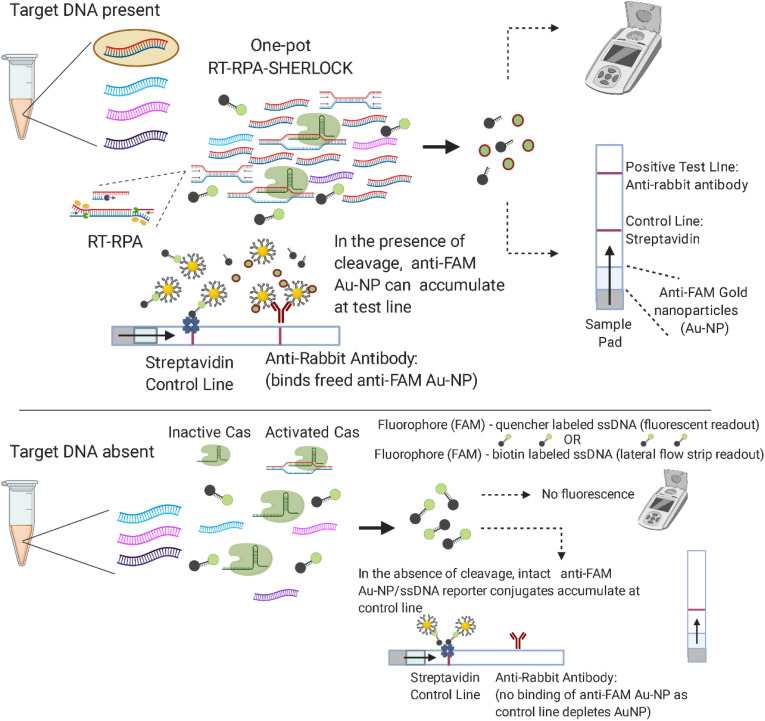
Schematic of one-pot SHERLOCK assay. RT-RPA amplifies *Plasmodium* species target sequences and occurs in parallel with programmed Cas12a detection, resulting in cleavage of target sequences and collateral cleavage of spiked fluorophore-labeled ssDNA reporter detectable by fluorescent measurement or lateral flow readout using Au-NP, gold nanoparticles.

Fig. 1 illustrates the workflow of our simplified SHERLOCK diagnostic. The test combines a 10-min sample preparation step and a 60-min SHERLOCK assay prior to endpoint analysis via lateral flow strip or fluorescence measurement. CRISPR-based diagnostics utilize the programmable endonucleases (Cas enzymes) of CRISPR-associated microbial adaptive immune systems. Cas12a (also known as Cpf1) is one such RNA-guided, DNA-cleaving enzyme, which can be programmed with CRISPR guide RNAs (gRNA) to construct highly sensitive and specific nucleic acid detection platforms (15–19). Programmed Cas12a is activated through recognition of its double-stranded DNA (dsDNA) target and exhibits indiscriminate, nonspecific DNase activity that cleaves nontarget DNAs. We exploit the nonspecific degradation of fluorophore-quencher labeled reporter single-stranded DNA (ssDNA) to detect the presence of the dsDNA target that activated Cas12a. To further decrease the LOD, a reverse-transcriptase recombinase polymerase amplification (RT-RPA) step is added before Cas12a detection to increase target DNA concentrations (Fig. 2). RPA is a powerful isothermal nucleic acid amplification tool comprised of three core enzymes: a recombinase, an ssDNA-binding protein, and a strand-displacing polymerase that coordinates DNA synthesis from primer-paired target DNA (20).

For endpoint analysis, released fluorophore from cleaved reporter ssDNA was measured by a plate reader or a handheld fluorimeter. Particularly in RLS, use of a handheld fluorimeter enables a field-applicable readout method. We did not find a significant difference in the sensitivity performance between machines and observed a similar 7- to 10-fold change in fluorescence between platforms, although they had different baselines (*SI Appendix*, Fig. S1). For use of the handheld fluorimeter, SHERLOCK reactions (50 μL) were performed combined in triplicate (150 μL) to increase the volume size for appropriate instrument reading.

Our assays are also adapted for endpoint detection via lateral flow strip based upon degradation of an ssDNA reporter that is labeled on opposing ends with FAM and biotin. The FAM-biotinylated reporter conjugates to anti-FAM gold nanoparticles contained within commercial lateral flow strips. If the reporter remains intact, FAM-labeled reporter/anti-FAM conjugates accumulate at the first line of the strip immobilized by streptavidin (control line). In the presence of activated Cas12a, the reporter is cleaved and freed FAM/anti-FAM conjugates are released to collect at the second line of the lateral flow strip containing anti-rabbit antibody (test line), which binds anti-FAM antibodies (Fig. 2).

There are many Cas enzymes that could have been used. We chose Cas12a [as opposed to the Cas13 family (21, 22), which also has nonspecific nuclease activity] so DNA targets could be directly detected instead of RNA, particularly in DBS where RNA may be degraded. The rapid enzymatic kinetics of Cas12a also make this nucleic acid-based technology comparable to the POC format of antigen-based lateral flow immunoassays. Cas12a bound to its dsDNA activator is capable of ∼1,250 turnovers per second with a catalytic efficiency (*k*_cat_/*K*_M_ ∼1.7 × 10^9^ s^−1^ M^−1^) approaching the rate of diffusion (17). The addition of a RT enzyme further enhances the sensitivity by transcribing multiple-copy RNAs from our target sequence into DNA for detection. We optimized SHERLOCK parameters, including reaction temperature, RPA primer concentration, RT commercial brand, and ssDNA reporter concentration (*SI Appendix*, Fig. S2). We also lyophilized the reaction into a pellet to be resuspended with an S-PREP–treated sample for cold-chain independence in the field, and importantly, also improved the LOD by increasing sample input volume.

### RPA Primer and gRNA Selection.

Our SHERLOCK assays were designed to detect four of the most common pathogenic species of malaria. We iterated a two-step design process of RPA primer screen followed by a gRNA screen. RPA primer targets were identified by reviewing the literature for the best-performing NAATs and searching for conserved and specific sequences from alignment of species-specific strains available from the National Center for Biotechnology Information (NCBI). For *P. falciparum* 18S rRNA, mitochondrial (cytochrome oxidase III, cytochrome B), and subtelomeric (Pfr364) targets were screened (23–29). The Pfr364 target, which is a species-specific, noncoding subtelomeric repeat sequence present in 41 copies on the *P. falciparum* genome, had the best signal in comparison to the other targets (*SI Appendix*, Fig. S3). Moreover, our selected gRNA had >90% sequence homology among all assembled *P. falciparum* genomes available in the NCBI, as well as 86% of sequences from the Pf3k dataset (an open-access collaboration and deep-genomic sequencing database) accessed via the Integrative Genomics Viewer (IGV) (30). For *P. vivax*, we tested an 18S rRNA and mitochondrial target, and found that the mitochondrial target worked best (copy number per parasite can be as high as 20) (27, 31). For *P. ovale* and *P. malariae*, we tested different regions of the 18S rRNA gene known to be conserved species-specific targets (27, 32, 33) typically present in four to eight copies per genome (notably, copy number is variable and depends on the parasite life cycle stage). We mapped the sequence targets’ primers and gRNA in *SI Appendix*, Fig. S4 and aligned them to the corresponding regions in off-target *Plasmodium* species (either homologous genes, or analogous sequences identified using NCBI’s Basic Local Alignment Search Tool [BLAST] with the lowest E-values). Despite overlap in RPA primers, which can tolerate significant sequence mismatch, we found that few-nucleotide differences in gRNA sequence were sufficient to obtain discriminating species-specific detection.

We constructed five forward (F1 to F5) and five reverse primers (R1 to R5) per sequence target using guidance provided by the TwistDx manufacturer; primers were 30 to 40 nucleotides long, with goal amplicons of 100 to 200 base pairs in length. We paired forward and reverse primers for a total of 25 combinations (F1:R1-5, F2:R1-5, F3:R1-5, F4:R1-5, F5:R1-5) for each sequence target and two to three of the best-performing pairs were selected for the optimization of gRNA design (*SI Appendix*, Fig. S5). RPA was performed according to the manufacturer’s instructions as described in *Materials and Methods*. Cas12a recognizes a short nucleotide sequence (TTTN) called the protospacer adjacent motif (PAM) for generation of distal dsDNA cleavage, and two to four gRNAs based upon the TTTN PAM were designed within the RPA amplicon. The RPA reaction for each primer set was then transferred to a Cas reaction as described in *Materials and Methods*, and fluorescent kinetics were monitored for selection of best-performing gRNAs (Table 1).

**Table 1.** Best-performing RPA primers and gRNA sequences for development of *Plasmodium* SHERLOCK assays

*Plasmodium* species: GenBank accession no. (sequence target)	RPA forward primer (5′ > 3′)	RPA reverse primer (5′ > 3′)	gRNA sequence (5′ > 3′)
*P. falciparum*	AAC​GCT​GCA​TTT​TGG​TCC​ATT​TTT​TGG​ACA​TTA​CG	TAA​AGG​AAC​AAT​TGC​CCC​ATG​TTT​TCC​CTG​CCC	GCG​CUA​AUA​CGA​CUC​ACU​AUA​GGG​UAA​UUU​CUA​CUA​AGU​GUA​GAU​AAA​ACA​UAA​GCG​UAG​AAA​CC
NC_004318.1			
(subtelomeric repeat)			
*P. vivax*	CCT​TAC​GTA​CTC​TAG​CTT​TTA​ACA​CAA​TAT​TAT​TGT​C	ACA​ATA​TTA​TAC​TGG​CAT​TTT​GTT​GAA​ATT​ATA​TGG​T	GCG​CUA​AUA​CGA​CUC​ACU​AUA​GGG​UAA​UUU​CUA​CUA​AGU​GUA​GAU​UAU​UCA​GAA​UAA​UGA​AUA​UA
JQ240387.1			
(mitochondrion)			
*P. ovale*	AAG​TTA​AGG​GAG​TGA​AGA​CGA​TCA​GAT​ACC​GTC​G	TAC​TCG​CCC​CAG​AAC​CCA​AAG​ACT​TTG​ATT​TCT​CAT​AAG​G	GCG​CUA​AUA​CGA​CUC​ACU​AUA​GGG​UAA​UUU​CUA​CUA​AGU​GUA​GAU​AAU​AAG​AAA​AUU​CCU​UUC​GG
AB182489.1			
(18S rRNA)			
*P. malariae*	AAC​GAA​AGT​TAA​GGG​AGT​GAA​GAC​GAT​CAG​ATA​CCG	TAC​TCG​CCC​CAG​AAC​CCA​AAG​ACT​TTG​ATT​TCT​CAT​AAG​G	GCG​CUA​AUA​CGA​CUC​ACU​AUA​GGG​UAA​UUU​CUA​CUA​AGU​GUA​GAU​UUU​UAG​AUA​GCU​UCC​UUC​AG
AF488000.1			
(18S rRNA)			

### Sample Preparation.

Accessing sample nucleic acids in a field-applicable manner involves overcoming several challenges. Preparation requires lysing the red blood cell (RBC) and parasite membrane (with the exception of the invasive merozoite form, all blood-stage parasites are intraerythrocytic), deactivating multiple inhibitory blood components, and importantly, appropriately deactivating nucleases that could shear the ssDNA reporter and lead to a false-positive signal. The requirement for simplicity and low cost ruled out commercial nucleic acid extraction kits. To test sample preparation methods, we used simulated whole-blood samples of live intraerythrocytic *P. falciparum* spiked into purchased EDTA-treated human blood (VWR International) to a final 1 fM (602 parasites per microliter) concentration for rehydration of our one-pot lyophilized *P. falciparum* SHERLOCK assay described in *Materials and Methods*.

One approach that did not work was HUDSON (heating unextracted diagnostic samples to obliterate nucleases), a simplified sample preparation method for viral nucleic acid extraction (16) compatible with Cas13 SHERLOCK. In HUDSON, whole-blood samples are pretreated with 100 mM TCEP [Tris(carboxyethyl)phosphine] and 1 mM EDTA to augment protein deactivation, followed by a two-step process of nuclease deactivation (heating for 5 min at 50 °C) followed by viral inactivation (heating for 5 min at 64 °C). HUDSON-treated simulated whole-blood samples produced minimal signal, likely from not accessing the intracellular parasitic nucleic acid.

**Fig. 3.**
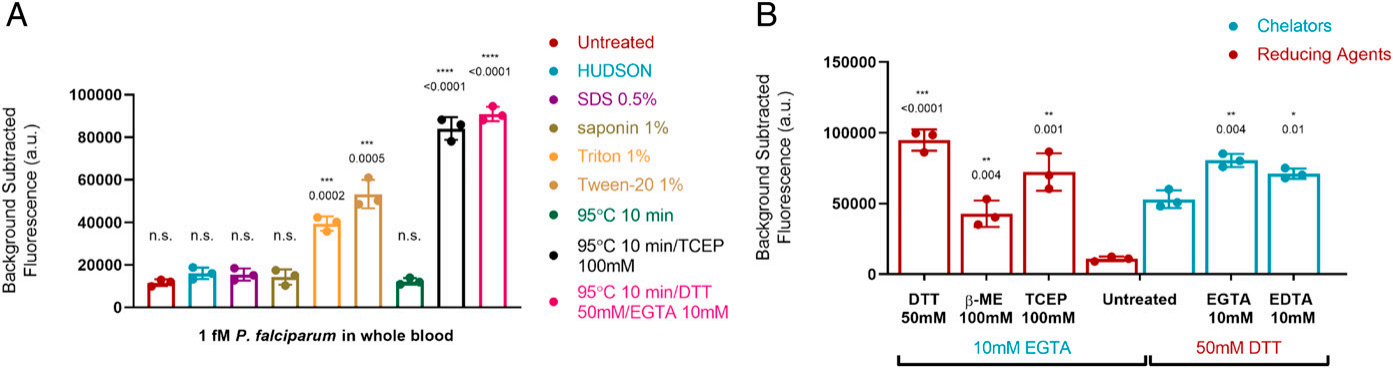
Sample preparation methods tested with SHERLOCK *P. falciparum* assay using simulated malaria samples of live intraerythrocytic *P. falciparum* spiked into whole blood at 1 fM (602 parasites per microliter) concentration. (*A*) Detergents and heating methods assessed for SHERLOCK compatibility. (*B*) Combinations of chelating and reducing agents tested for optimization of chemical deactivation of nucleases and inhibitors. Asterisks indicate significant differences from untreated simulated whole blood sample assessed by Student’s two-tailed *t* test. Bars: mean ± SD of three technical replicates. *P < 0.05, **P < 0.01, ***P < 0.001, ****P < 0.0001, ns, not significant.

**Fig. 4.**
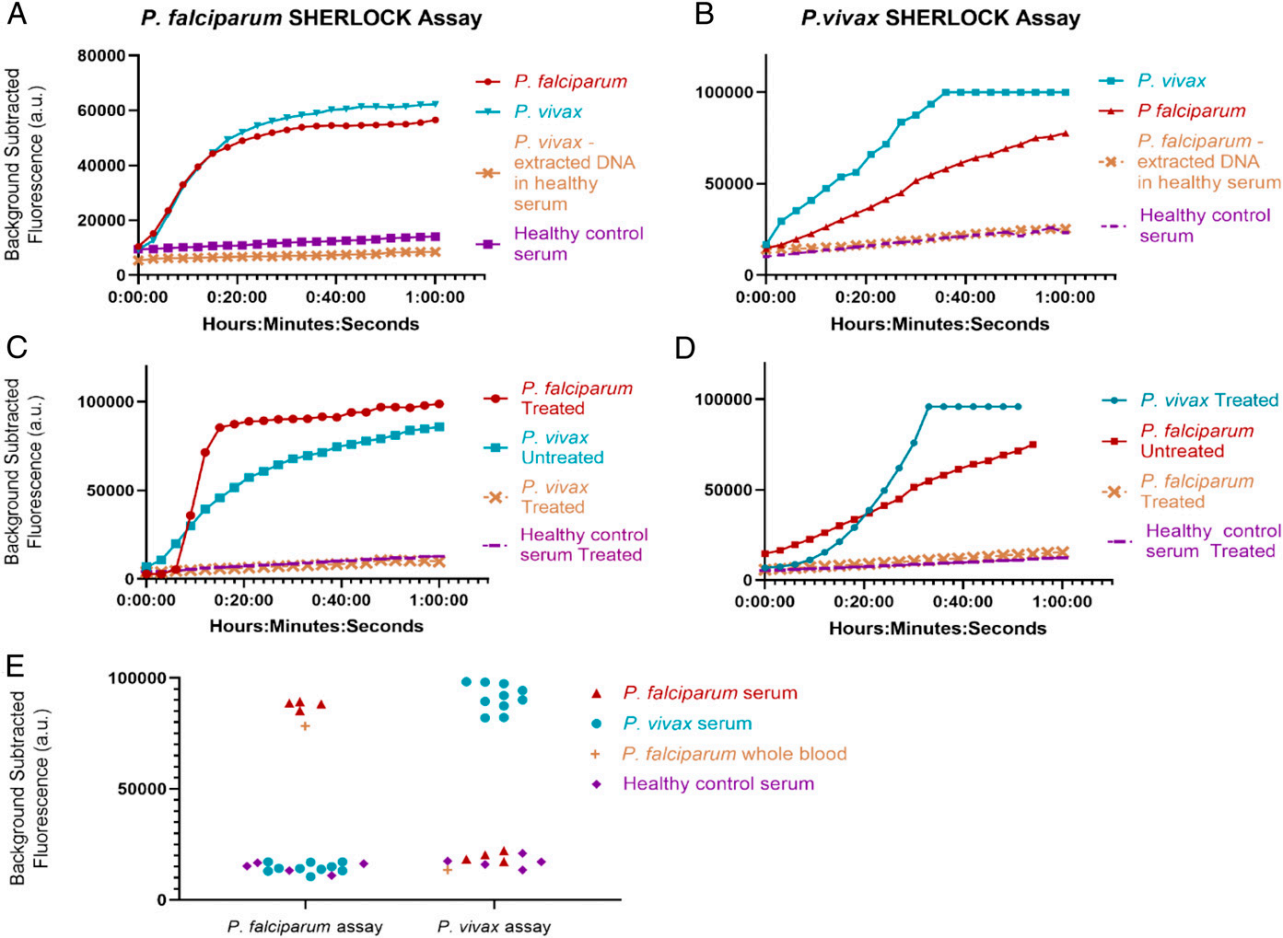
Specificity of SHERLOCK assays. (*A*) Using *P. falciparum* assay and DTT/EGTA/95 °C sample preparation, *P. falciparum* and *P. vivax* patient serum in SHERLOCK diagnostic display similar fluorescent kinetics that are eliminated when an aliquot of the same *P. vivax* serum undergoes nucleic acid extraction via commercial kit. (*B*) Using *P. vivax* assay and DTT/EGTA/95 °C sample preparation, *P. falciparum* serum demonstrates a false-positive signal that is eliminated when an aliquot of the same *P. falciparum* serum undergoes nucleic acid extraction via commercial kit. (*C*) False-positive *P. vivax* signal is eliminated with S-PREP. (*D*) False-positive *P. falciparum* signal is eliminated with S-PREP. (*E*) Performance of SHERLOCK diagnostic on clinical patient serum and whole-blood samples prepared with S-PREP: 5 *P. falciparum* samples (4 serum, 1 whole blood), 10 *P. vivax* serum samples, and 5 serum samples from healthy controls.

We therefore assessed alternative simplified sample preparation protocols described in *Materials and Methods*, including various detergents, thermal lysis, and chemical deactivation protocols (Fig. 3). We discovered that treating samples with 50 mM DTT and 10 mM EGTA, followed by 95 °C incubation for 10 min resulted in a robust SHERLOCK signal, although we noticed variability in the no-template control signal that we attributed to background nucleases in different blood aliquots. However, when we tested the DTT/EGTA/95 °C sample preparation method on patient *P. falciparum* and *P. vivax* serum samples from the Dominican Republic, we found bidirectional cross-reactivity of our species-specific SHERLOCK assays. Using our *P. falciparum*-specific assay, *P. vivax* patient serum samples produced a false-positive signal (Fig. 4*A*). *P. falciparum* patient samples also produced a false-positive signal using the *P. vivax-*specific assay (Fig. 4*B*).

The false-positive signals were eliminated, however, when DNA from the same cross-reacting *P. vivax* and *P. falciparum* patient serum samples was extracted via QIAamp DNA mini kit (Qiagen), spiked into a healthy commercial serum no-template control (10-ng extracted DNA into 20 µL serum; Sigma Aldrich), and retested (Fig. 4 *A* and *B*). Furthermore, the extracted patient serum DNA maintained a robust species-specific signal with the appropriate *Plasmodium* species-specific assay. Extracted nucleic acid reflected combined human and parasite DNA, with numbers of human sequences dwarfing numbers of parasite sequences, and the highly sensitive and specific performance of the appropriate SHERLOCK assay on the extracted nucleic acid made cross-reactivity due to human DNA unlikely. These results were also observed on all 5 *P. falciparum* and all 10 *P. vivax* specimens, making coinfection unlikely and the specimens had all undergone species-specific qualitative PCR testing (ARUP). We hypothesized that the cross-reactivity could be secondary to nonspecific ssDNA reporter cleavage from higher concentrations of nucleases in “sick” versus “healthy” serum that resulted in incomplete deactivation of nucleases in “sick serum” by our DTT/EGTA/95 °C simplified preparation method.

This hypothesis was confirmed when we developed S-PREP using a buffer comprised of a stronger chelating agent: 20% (wt/vol) Chelex-100 (Bio-Rad) suspended in TE buffer with 50 mM DTT. Chelex-100 is a resin containing styrene divinylbenzene copolymers with paired iminodiacetate ions that act as chelating groups in binding polyvalent metal ions (34). Nucleases require metal ions as cofactors and therefore chelating agents inhibit their activity. S-PREP is a simplified sample preparation method where sample is diluted 1:3 (5 μL into 15 μL of S-PREP buffer) followed by heating to 95 °C for 10 min. We eliminated the false-positive signals of serum samples using S-PREP (Fig. 4 *C* and *D*). We conclude that higher concentrations of nucleases present in “sick” serum (patients sick with another disease but not the target disease) necessitate stronger nuclease deactivation procedures. We are unique in reporting on this cross-reactivity in nonnucleic-acid extracted clinical samples for SHERLOCK, as we are not aware of other studies comparing performance using unextracted samples against controls from patients sick with a different disease (instead of only comparing to healthy control specimens). This highlights the importance of considering baseline nuclease activity in specimen types with CRISPR-based assays, as the readout is dependent on reporter nucleic acid cleavage and contaminating nucleases are a major concern for false positives. Importantly, we demonstrated that S-PREP can deactivate high levels of nucleases and we confirmed the absence of false positives in our clinical sample set with 100% specificity (*P. falciparum n* = 4 serum, *n* = 1 whole blood; *P. vivax n* = 10 serum; healthy serum patient controls *n* = 5) (Fig. 4*E*). We additionally found that S-PREP was compatible with RNA-only simulated samples, despite its increased susceptibility to hydrolysis in comparison to DNA, demonstrated by detection of an RNA-only synthetic target prepared using S-PREP (SARS-CoV-2 RNA target detected in novel SHERLOCK assay) (*SI Appendix*, Fig. S6).

To further assess the field versatility of our work, we also tested our S-PREP/SHERLOCK diagnostic on simulated samples from multiple specimen collection types. We spiked live intraerythrocytic *P. falciparum* into whole blood and plasma stored in multiple different specimen collection tubes (acid-citrate dextrose, EDTA K-2, EDTA K-3, Na heparin, Na citrate, plasma heparin, and plasma EDTA) to a final 1 fM (602 parasites per microliter) concentration and prepared these samples with S-PREP for rehydration of our SHERLOCK assay, as described in *Materials and Methods*. Although many of these additives are known PCR inhibitors, all simulated samples were able to produce a distinguishable signal from the no-template control (*SI Appendix*, Fig. S7). The compatibility of SHERLOCK with unextracted samples from multiple specimen tube types emphasizes its unique robustness, versatility, and ultimately suitability for RLS.

### Performance and Readout of Malaria SHERLOCK Diagnostic.

**Fig. 5.**
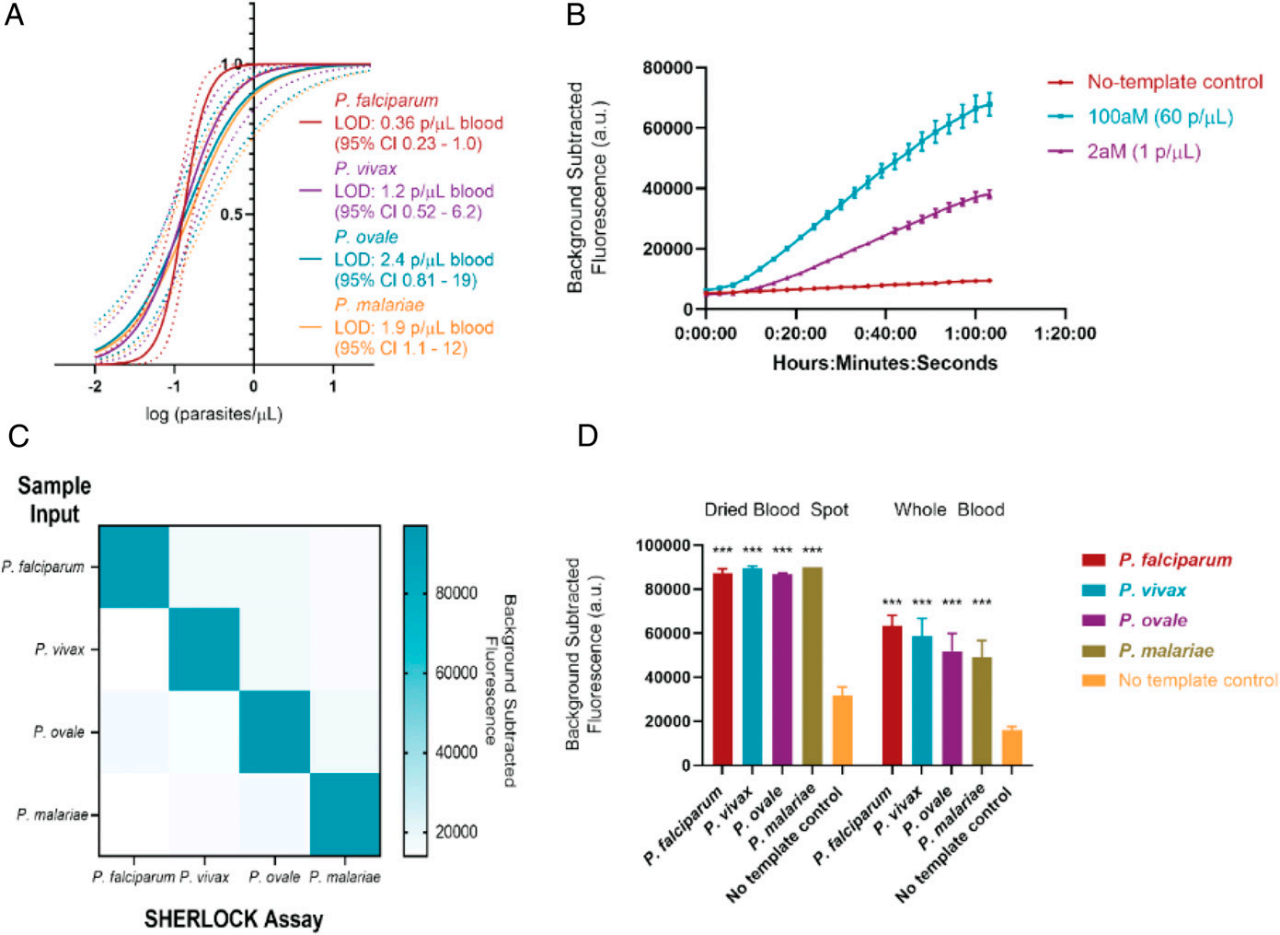
SHERLOCK performance. (*A*) Sensitivity of SHERLOCK diagnostic for detection of *Plasmodium* species by comparison of probit regression curves obtained from 21 replicates of 5 dilutions. (*B*) Fluorescence kinetics of *P. falciparum* SHERLOCK assay at 100 aM (60 parasites per microliter) and 2 aM (1 parasite per microliter) concentrations. (*C*) Specificity of SHERLOCK diagnostic using 10 fM (6,020 parasites per microliter) concentrations of parasite. (*D*) Comparison of performance between simulated DBS and whole-blood samples. All experiments used simulated whole-blood samples. ***P < 0.001 for Student’s *t* test between fluorescent output of sample type versus no-template control.

We determined the analytical sensitivity of our assays using industry standard definitions of the diagnostic LOD to guarantee a 95% probability of successful detection. We performed septet replicate testing on three different runs on simulated whole-blood samples (described in *Materials and Methods*) for each *Plasmodium* species and used probit analysis to establish: *P. falciparum* 0.36 parasite per microliter blood (95% confidence interval [CI] 0.23 to 1.0), *P. vivax* 1.2 parasites per microliter (95% CI 0.52 to 6.2), *P. ovale* 2.4 parasites per microliter (95% CI 0.81 to 19), and *P. malariae* 1.9 parasite per microliter (95% CI 1.1 to 12) (Fig. 5*A* and Table 2). This reaches the WHO LOD goal for low endemnicity (asymptomatic carriage) settings and is notable in that SHERLOCK was capable of attomolar to subattomolar detection in the absence of commercial kit nucleic acid extraction and sample nucleic acid concentration. This also emphasizes the ultrasensitive capacity of SHERLOCK in that our best detection level (0.36 parasite per microliter for *P. falciparum*) closes in on the theoretical LOD of the engineering design. Using 12.5 µL of sample input as we have, a 0.3 parasite per microliter concentration sample has a 95% probability of containing at least one parasite (and therefore being detectable) following the Poisson distribution (*SI Appendix*, Fig. S8).

**Table 2.** Analytical sensitivity of *Plasmodium* species SHERLOCK

	95% LOD p/µL (95% CI)	50 zM (0.03 p/µL)	200 zM (0.12 p/µL)	500 zM (0.3 p/µL),	5 aM (3 p/µL),	50 aM (30 p/µL).
*P. falciparum*	0.36 (0.23–1.0)	0/21	10/21	19/21	21/21	21/21
*P. vivax*	1.2 (0.52–6.2)	1/21	13/21	16/21	20/21	21/21
*P. ovale*	2.4 (0.81–19)	1/21	15/21	14/21	19/21	21/21
*P. malariae*	1.9 (1.1–12)	0/21	13/21	16/21	18/21	21/21

Results of replicate testing at five different calibration standard concentrations near the expected LOD (replicates testing positive/replicates tested for determination of 95% LOD by probit analysis); p/µL, parasites per microliter in contrived calibration sample (prior to S-PREP dilution).

Our CRISPR diagnostic can also detect clinically relevant levels of parasitemia in 40 min or less from unextracted blood samples (10-min S-PREP followed by 30-min SHERLOCK) with better sensitivity than existing POC antigen-based RDTs, filling an important clinical diagnostic gap for *hrp2* deletion *P. falciparum* and nonfalciparum malaria. A 0.001% parasitemia (assuming a RBC mean corpuscular volume of 80 fL and hematocrit of 45%) corresponds to ∼60 parasites per microliter (100-aM concentration), for which a 30-min detectable signal difference between the no-template control and infected blood is readily apparent (Fig. 5*B*). This level of parasitemia would likely be missed on RDT or light microscopy (a technician would have to view 100,000 RBCs to view an infected RBC, which is theoretically possible, but would require considerable effort). Finally, while there is no consensus definition of asymptomatic malaria, some have used parasite density cutoffs of 5,000 parasites per microliter blood (∼8.5 fM) as a threshold (vaccine trials and epidemiological studies), which is a rapidly detectable concentration with SHERLOCK (35–37).

The analytical specificity of our assays was determined using simulated clinical samples at a 10-fM concentration (6,020 parasites per microliter) and demonstrated no detection of nontarget *Plasmodium* species, confirming high specificity (Fig. 5*C*). We surmise that the highly specific performance of these assays is likely attributable to a two-step target selection via RPA primer match and amplification, followed by gRNA match and Cas activation. For clinical sensitivity and specificity, we were able to detect and differentiate 5 *P. falciparum* (4 serum, 1 whole blood) and 10 *P. vivax* samples with 100% accuracy (Fig. 4*E*). Deidentified clinical samples were purchased from BocaBiolistics and came from symptomatic patients from the Dominican Republic. They had been previously characterized by both the BinaxNOW Malaria RDT (Alere) and species-specific qualitative PCR (ARUP), demonstrating that our diagnostic had 100% concordance with these methods in our limited clinical set.

We additionally prepared simulated DBS to a 2-aM (one parasite per microliter blood) concentration of the four *Plasmodium* species and tested them with our S-PREP/SHERLOCK protocol with modifications, as described in *Materials and Methods*. A robust fluorescence signal was demonstrated at the 1-h time point that was significantly different from the no-template control. The only notable difference in assay performance compared with whole-blood samples was a greater no-template control signal in simulated DBS samples, likely from autofluorescence from the paper substrate (Fig. 5*D*).

**Fig. 6.**
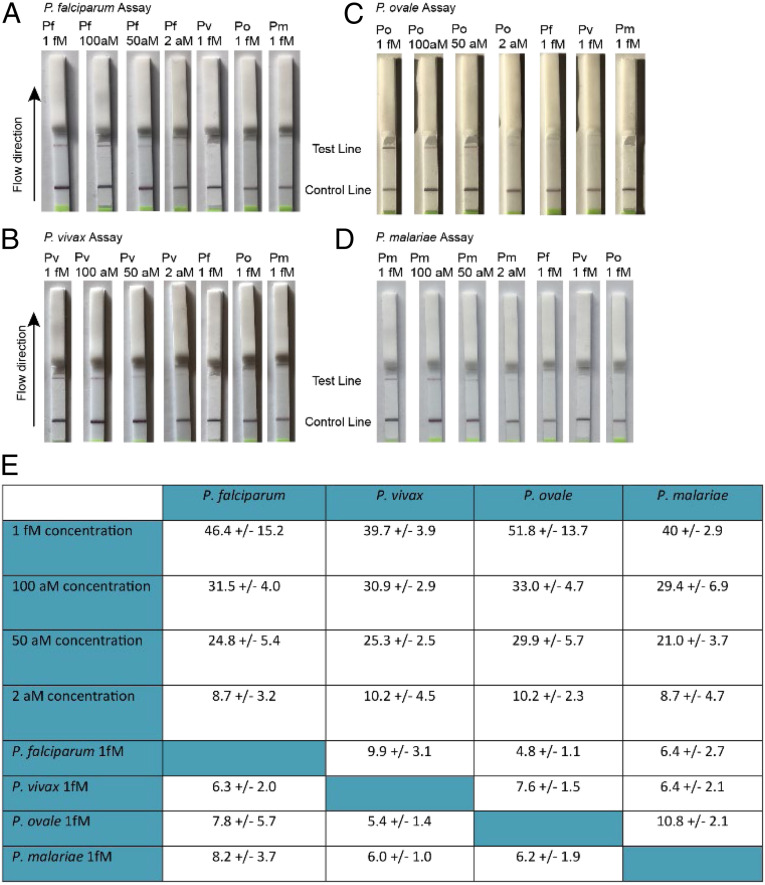
SHERLOCK lateral flow assay performance. (*A*–*D*) Detection of 1 fM (∼602 parasites per microliter), 100 aM (60 parasites per microliter), 50 aM (30 parasites per microliter), and 2 aM (1 parasite per microliter) concentrations of *P. falciparum, P. vivax*, *P. ovale*, and *P. malariae*, respectively, and comparison to 1-fM concentrations of off-target *Plasmodium* species for each assay. (*E*) Background-subtracted grayscale intensity averages of test line for three separate flow tests ±SD. All experiments used simulated whole-blood samples.

In addition to establishing the analytical LOD via fluorescent measurement, we also demonstrated a lateral flow readout given its ease of use in RLS. We found that a clearly visible band was distinguishable at 50 aM (30 parasites per microliter) for all of the *Plasmodium* species assays (Fig. 6); this LOD is higher than that of our fluorescent readout, but it is still lower than best-in-class contemporary RDTs (38).

## Discussion

We demonstrated a simplified SHERLOCK diagnostic comprised of a 10-min S-PREP followed by SHERLOCK for *Plasmodium* species-specific detection via fluorescent or lateral flow strip readout. Our advancements could fill significant gaps in malaria diagnostics by establishing a field-applicable diagnostic for ultrasensitive detection of asymptomatic carriers and malaria eradication, and a POC clinical diagnostic for *hrp2* deletion *P. falciparum* infections and nonfalciparum malaria species. This is a particularly important goal for *P. vivax*, the most widely distributed malaria pathogen worldwide, missed by many contemporary RDTs and requiring different therapy than *P. falciparum.*

We rigorously optimized our assays for field implementation. We demonstrated a fully lyophilized one-pot SHERLOCK protocol on clinical samples only requiring rehydration of the reaction with the sample, eliminating the labor and contamination risk of multiple specimen transfer steps. Lyophilization also enables cold-chain independence, and improves the LOD by increasing sample input volume (12.5-µL vs. 4.25-µL blood input in a nonlyophilized reaction).

These results highlight the applicability of SHERLOCK platforms to the arena of global health and RLS. SHERLOCK is a cost-effective technology estimated at $0.61 (USD) per test (15), given its lyophilizable format and lateral flow readout capability. Our work brings the platform closer to clinical care in demonstrating a field-ready SHERLOCK diagnostic. Key features include simplified sample preparation without nucleic acid extraction, isothermal assay conditions (40 °C) independent of a thermocycler, a lyophilized integrated assay, and field-applicable readouts, including use of a handheld fluorimeter or lateral flow strip. We further validated the ultrasensitive LOD of our assays using industry standard protocols of replicate testing.

We additionally gained critical insight into engineering design considerations for ultrasensitive microvolume and SHERLOCK-based diagnostics. First, we highlighted an underappreciated concept that when reaching attomolar and subattomolar concentrations where assays are capable of one copy per assay detection levels, the rate-limiting consideration is the probability of pathogen presence in the sample input volume, no longer approximated by a Gaussian but instead Poisson distribution, as the probability of blank inputs is significant. For the cumulative distribution function to reach 100% for a 2-aM pathogen concentration (guaranteeing at least one target copy in the sample volume), the sample input volume must be at least 12 μL (*SI Appendix*, Fig. S8). Second, we demonstrated that a key limitation of SHERLOCK assays, in general, is that their readout dependence on ssDNA cleavage makes the assays highly susceptible to false positives in the presence of contaminating nucleases. While all NAATs are at risk for target degradation in the presence of nucleases, appropriate deactivation is crucially important for SHERLOCK assays, and we observed that specimens may very well have differing levels of nucleases depending on disease state, sample type, and even blood aliquot.

Limitations of our study include a limited clinical validation sample set, and we are moving forward with plans for obtaining larger specimen sets, including whole-blood asymptomatic patient samples and *P. malariae* and *P. ovale* patient samples. Whole blood is also a more common specimen type than serum for *Plasmodium* detection, given the intraerythrocytic location of parasites (*Plasmodium* nucleic acid is likely present in residual amounts in serum); however, we were only able to purchase mostly serum samples. Notably, the robust performance of our diagnostic on lower pathogen-load serum samples demonstrates the high sensitivity of SHERLOCK. Additionally, while our lateral flow assay LOD (30 parasites per microliter) was higher than that of the fluorescent readout, we expect that the lateral flow format is more relevant for clinical diagnosis versus asymptomatic mass screening, which is more amenable to batch testing in a plate reader. This LOD is nevertheless an order-of-magnitude lower than the 200 parasites per microliter “low parasite density” testing threshold used in the WHO’s latest Malaria Rapid Diagnostic Test Performance report (38). Future work will need to further optimize the lateral flow assay.

Notably, as diagnostics become increasingly capable of ultrasensitive limits of detection, it is important to consider whether technologies may detect pathogens below the level of clinical and epidemiological relevance. Future studies will also be needed to better characterize the bloodstream clearance kinetics of ultralow parasitemia. Currently, it is unknown whether trace amounts of DNA may persist for several days after treatment or prophylactic therapy, and falsely raise concerns of drug failure. Furthermore, while evidence suggests that asymptomatic carriers are likely contributing to ongoing spread of malaria (2), it is unclear if there is a pathogen burden cutoff below which transmission is unlikely.

In summary, our malaria SHERLOCK diagnostic for ultrasensitive and specific *Plasmodium* species identification is a promising tool that moves this technology closer to clinical POC application in resource-limited settings. Future work will be needed to understand performance in field settings and define the utilization of ultrasensitive detection for clinical and policy decision making.

## Materials and Methods

### Simulated Samples and Clinical Samples.

*P. falciparum*-simulated samples were prepared by either serially diluting live parasites into whole blood or serially diluting purified whole-genomic DNA into whole blood. To prepare simulated infected whole blood with live intraerythrocytic *P. falciparum*, the 3D7 strain (obtained from the Walter & Eliza Hall Institute of Medical Research, Parkville, Australia) of *P. falciparum* was cultured in human RBCs at 4% hematocrit to ∼2% parasitemia in RPMI 1640 supplemented with 0.5% Albumax II, 50 mg/L hypoxanthine, 0.21% sodium bicarbonate, and 25 mM Hepes, as previously described (39). Aliquots of cultures with known parasitemia (parasites per microliter RPMI 1640) determined by microscopy via triplicate field-stained blood smears with average parasitemia calculated were spiked into uninfected whole blood (VWR International) stored with EDTA anticoagulant to make serial dilutions. For LOD calculations, extracted whole-genomic DNA harvested from cultured *P. falciparum* via QIAamp Blood Mini Kit (Qiagen) was quantified (ng/μL) on the NanoDrop 2000 (Thermo Fisher Scientific), and spiked into uninfected whole blood or serum and serially diluted. Molar concentration was calculated by the estimated molecular weight of a 22.8-Mb genome (40). dsDNA molecular weight can be estimated from genome size by multiplying the number of base pairs of dsDNA by the average molecular weight of a base pair (650 g/mol) (41). The molar concentration calculated by dividing the mass of a sample by its molecular weight can be translated to copies of target (parasites) per unit volume by multiplying by Avogadro’s number (6.022 × 10^23^ molecules/mole).

For *P. vivax*, we extracted nucleic acid from patient clinical samples via a QIAamp Blood Mini Kit, measured DNA concentration via Nanodrop, and used the estimated molecular weight presuming a 26.8-Mb genome (42) to calculate a molar concentration. Serial dilutions of concentrated DNA into whole blood were used for LOD measurements (Fig. 5).

For *P. malariae* and *P. ovale*, we obtained plasmids containing the small subunit ribosomal RNA genes (18S) MRA-179 and MRA-180, contributed by Peter A. Zimmerman from BEI Resources, National Institute of Allergy and Infectious Diseases, NIH, Bethesda, MD. After quantification of plasmid on Nanodrop and using the estimated molecular weight based on known plasmid size (5,100 base pairs and 5,000 base pairs, respectively) for calculation of molar concentration, we serially diluted plasmids into whole blood to determine the LOD (Fig. 5).

DBS were simulated by deposition of 50 µL of simulated blood samples (live intracellular *P. falciparum* spiked into whole blood, *P. vivax* purified whole genomic DNA spiked into whole blood, *P. malariae* MRA-179 plasmid spiked into whole blood, *P. ovale* MRA-180 plasmid spiked into whole blood) ×2 onto Whatman 903 Protein saver cards (Thermo Fisher Scientific). The DBS were dried in ambient conditions for 3 h and then tested as described below in the sample preparation and SHERLOCK reaction procedure.

Four serum (collected in serum separator tubes), and 1 whole-blood (collected in K2-EDTA tube) *P. falciparum* and 10 serum (collected in serum separator tubes) *P. vivax* samples from deidentified symptomatic patients in the Dominican Republic were purchased from BocaBiolistics. Samples had been previously characterized by Alere BinaxNOW Malaria RDT and qualitative species-specific PCR (ARUP). All clinical samples and human RBC aliquots used had been previously deidentified prior to purchase.

### RPA Primer, gRNA Screen, and Construction.

Conserved *Plasmodium* regions identified from the literature and publicly accessible databases (NCBI, Pf3k, and PlasmoDB) were used to generate target RPA primers and gRNA sequences. Alignments to ensure conservation of targets across available individual species’ genome assemblies, as well as exclusivity between *Plasmodium* species, were performed using MAFFT (43) and visualized with Jalview 2.11.1.0 (44). RPA primers were purchased from Integrated DNA Technologies (IDT). The CRISPR gRNA was produced by in vitro transcription from synthetic DNA sequences purchased from IDT using the HiScribe T7 Quick High Yield RNA Synthesis kit (New England Biolabs) and purified using the RNA Clean and Concentrator kit (Zymo Research). A quenched fluorescent ssDNA reporter with a 5′ end-labeled FAM group and a 3′ end attached to an Iowa Black quencher (56-FAM/TTATT/3IABkFQ) was purchased from IDT. RPA primer screens were conducted using 7.5-µL reaction volumes of RPA basic kit (TwistDx) spiked with unique primer sets to final concentrations as recommended per the manufacturer’s instructions: 14 mM magnesium acetate, 490 µM RPA primers each, and 0.6× rehydration buffer incubated at 40 °C for 30 min. Initial screen gRNAs were constructed for expected RPA amplicons of different sequence targets. Collateral degradation of ssDNA reporter upon Cas12a activation was measured by mixing 2 µL of a RPA primer screen reaction into a 10-µL reaction volume with final concentrations of 100 nM Cas12a (New England Biolabs), 200 nM gRNA, 1× NEB 2.1 buffer (New England Biolabs), and 1 µM ssDNA reporter. We incubated the mixture at 40 °C for 120 min and measured fluorescence kinetics in a BioTek NEO HTS plate reader (BioTek Instruments) with readings every 3 min (excitation: 485 nm; emission: 535 nm). Best-performing RPA primer sets from sequence targets were selected for testing of two to three gRNAs constructed from the RPA amplicon region, using the same protocol.

### Sample Preparation Testing.

Using live intraerythrocytic *P. falciparum* spiked into whole blood as a simulated malaria sample, we trialed multiple sample preparation methods. All sample preparation methods tested had a final volume of 20 μL with a final *P. falciparum* concentration of 1 fM or 602 copies per microliter (various methods had different dilution steps and so initial spiked concentration varied) and were tested via rehydration of the one-pot lyophilized SHERLOCK *P. falciparum* pellet described below. Fluorescence was measured over 1 h at 40 °C using a BioTek NEO HTS plate reader with readings every 3 min (excitation: 485 nm; emission: 535 nm). Detergents at varying wt/vol% (SDS 0.5%, saponin 1%, Tween-20 1%, Triton-X 100 1%) were added to a 20-μL simulated whole-blood sample along with 100 mM TCEP. Two heating sample preparation protocols were tested: 1) dilution of simulated sample 1:4 in nuclease-free water followed by 10-min 95 °C incubation (1:4 dilution required to prevent solidification when diluting with water), and 2) addition of 100 mM TCEP into the diluted simulated sample prior to 10-min 95 °C incubation. For optimization of chemical deactivation methods of nucleases and SHERLOCK inhibitors, combinations of chelators and reducing agents added to 20-μL simulated samples at concentrations demonstrated in Fig. 3*B* were tested.

### S-PREP Sample Preparation.

Inactivation (nucleases and inhibitors) of whole-blood and serum samples was performed by dilution of sample in 1:3 ratio (12.5-µL sample: 37.5-µL S-PREP buffer); S-PREP buffer consisted of Tris-EDTA buffer (Invitrogen) with 50 mM DTT (Sigma Aldrich) and 20% (wt/vol) Chelex-100 (Bio-Rad). Samples were then heated to 95 °C for 10 min. For simulated DBS, a disposable biopsy punch (VWR International) was used to make 2-mm-diameter disks from DBS-simulated samples that were dropped into 200-µL PCR-compatible tubes. Then, 50 µL of S-PREP buffer was added to the tube followed by 95 °C heat inactivation for 10 min. For testing of compatibility of S-PREP and SHERLOCK with different collection tube types, live intraerythrocytic *P. falciparum* spiked into whole blood collected from different collection tubes to a final 1-fM (602 parasites/µL) concentration was prepared via S-PREP (5-μL simulated sample into 15-μL S-PREP buffer followed by 10 min 95 °C heating) and used to rehydrate the SHERLOCK lyophilized reaction described below. To demonstrate compatibility of S-PREP with RNA, synthetic SARS-CoV-2 RNA SKU 103086 (Twist Bioscence) was prepared using S-PREP and tested in SARS-CoV-2 SHERLOCK assay in development.

### Preparation of Lyophilized SHERLOCK Reactions and Procedure.

SHERLOCK reactions were prepared to 50 µL using 100 nM Cas12a, 200 nM gRNA, 0.8× NEB buffer 2.1, 430 nM of each RPA primer, 2 U/µL ProtoScript II reverse-transcriptase (New England Biolabs), 0.6× RPA rehydration buffer, 14 mM MgOAc, 10 mM EGTA, and 1 µM FAM-Iowa Black quenched ssDNA fluorescent reporter. For lateral flow readout, 1 µM fluorophore-biotin–labeled ssDNA reporter (56-FAM/TTATT/3Bio; IDT) was used instead of fluorophore-quencher reporter.

Reactions were prepared in 200-µL PCR-compatible tubes and a small opening was pierced in the cap with a 25-gauge × 5/8 (0.5 mm × 16 mm) BD PrecisionGlide Needle (Becton Dickinson) to allow for sublimation during lyophilization. Reaction tubes were placed in a chilled metallic tube rack and submerged for 1 min in liquid nitrogen. The snap-frozen tubes and rack were wrapped in Kimwipes (Kimberly-Clark) and three layers of aluminum foil. The entire bundle was then placed inside a sealed glass lyophilization chamber and connected to a freeze-drying machine (Labconco). Lyophilization was performed for 6 h. Activation of reaction was performed by rehydration in 50 µL of sample prepared by S-PREP (12.5 µL of sample into 37.5 µL of buffer followed by 95 °C incubation). Notably, for testing of simplified sample preparation methods, lyophilization reactions were scaled to a 20-μL sample input volume, so 20-μL SHERLOCK reactions were lyophilized and 20 μL of simulated sample prepared by tested preparation methods were used for rehydration of reaction. Fluorescence was measured over 1 to 3 h at 40 °C using a BioTek NEO HTS plate reader with readings every 3 min (excitation: 485 nm; emission: 535 nm). For field simulation, a start and 1-h fluorescence measurement were made with a Quantus fluorimeter (due to a minimum volume instrument input, the reaction was performed in triplicate, although could have been diluted, albeit with lower signal output). For DBS assays, the supernatant from the DBS/S-PREP reaction was transferred to lyophilized SHERLOCK pellets for resuspension of reaction; the 2-mm DBS punch and resuspended SHERLOCK reactions were then transferred to a 384-well plate for fluorescence measurement by same protocol as non-DBS reactions. For lateral flow readout, 20 µL of the SHERLOCK endpoint reaction was added to 100 µL of HybriDetect 1 assay buffer and run on HybriDetect 1 lateral flow strips (Millenia).

### Clinical and Analytical Specificity of Patient Serum Samples.

For demonstration of specificity on clinical samples (*P. falciparum n* = 4 serum, *n* = 1 whole blood; *P. vivax n* = 10), 12.5 µL of serum (or whole blood) was diluted into 37.5 µL S-PREP buffer (20% [wt/vol] Chelex-100 in TE buffer with 50 mM DTT). For determination of analytical specificity, three replicates of *P. falciparum*, *P. vivax*, *P. ovale*, and *P. malariae* simulated whole-blood samples were prepared to a final concentration of 10 fM (6,020 parasites per microliter) as described above and similarly diluted in S-PREP buffer. Prepared simulated or real patient samples were then incubated at 95 °C for 10 min and transferred to a SHERLOCK lyophilized pellet, as described above, for resuspension of reaction. Fluorescence was measured over 1 h at 40 °C using a BioTek NEO HTS plate reader with readings every 3 min (excitation: 485 nm; emission: 535 nm).

### Determination of Analytical Sensitivity, LOD.

The analytical LOD was defined as the lowest *Plasmodium* species concentration that was successfully detected with a probability of 95% or greater. Calibration standards near the estimated LOD were prepared by serial dilutions of simulated samples described above to the following concentrations: 50 zM (0.03 copies per microliter sample), 200 zM (0.12 copies per microliter), 500 zM (0.3 copies per microliter), 5 aM (3 copies per microliter), 50 aM (30 copies per microliter). The LOD was evaluated by testing the calibration standard over 3 separate runs performed on different days with 7 replicates for each concentration, for a total of 21 replicate results at each concentration level.

### Data Analysis.

Background-subtracted fluorescence was calculated by subtraction of the fluorescence of no-input (water only as “template” input into SHERLOCK reaction) control wells on the plate from target fluorescence values evaluated in the assay run at the same time points in the assay. Water-only control wells were therefore subtracted from both no-template controls (such as whole blood or serum) and samples or simulated sample wells. Student’s *t* tests were used for comparison of background-subtracted fluorescence between *Plasmodium* targets and controls. A *P* value of < 0.05 was considered statistically significant. The relationship between the proportion of replicates testing positive and the corresponding sensitivity standard *Plasmodium* log concentration was examined using Probit regression analysis to estimate 95% LOD and 95% CI of each target (GraphPad 8.4.1). Lateral flow test line signal intensities were quantified to grayscale pixel values using ImageJ software (National Institutes of Health). Background-subtracted intensity was calculated from line scans that spanned the 1-mm test line subtracted from background blank (white) area to normalize to ambient background grayscale value of the lateral flow strip.

## Supplementary Material

Supplementary File

## Data Availability

All study data are included in the article and *SI Appendix*.
