# Supplementary material for: Ultrasensitive CRISPR-based diagnostic for field-applicable detection of Plasmodium species in symptomatic and asymptomatic malaria
Source: Proc Natl Acad Sci U S A. 2020 Sep 21;117(41):25722–31. doi: 10.1073/pnas.2010196117 (PMC7568265; doi:10.1073/pnas.2010196117)
Supplement: Supplementary File [file pnas.2010196117.sapp.pdf]

PNAS
www.pnas.org

Supplementary Information for

Ultrasensitive CRISPR-based Diagnostic for Field-applicable Detection of *Plasmodium* Species in Symptomatic and Asymptomatic Malaria

Rose A. Lee[1,4,5], Helena De Puig[1,2,], Peter Q. Nguyen[1,8], Nicolaas Angenent-Mari[1,2], Nina M. Donghia[1], James P. McGee[4], Jeffrey D. Dvorin[4], Catherine M. Klapperich[6], Nira R. Pollock[5,7], and James J. Collins[1,2,3]

*James J. Collins
email: jimjc@mit.edu

This PDF file includes:

Figures S1 to S8

www.pnas.org/cgi/doi/10.1073/pnas.2010196117

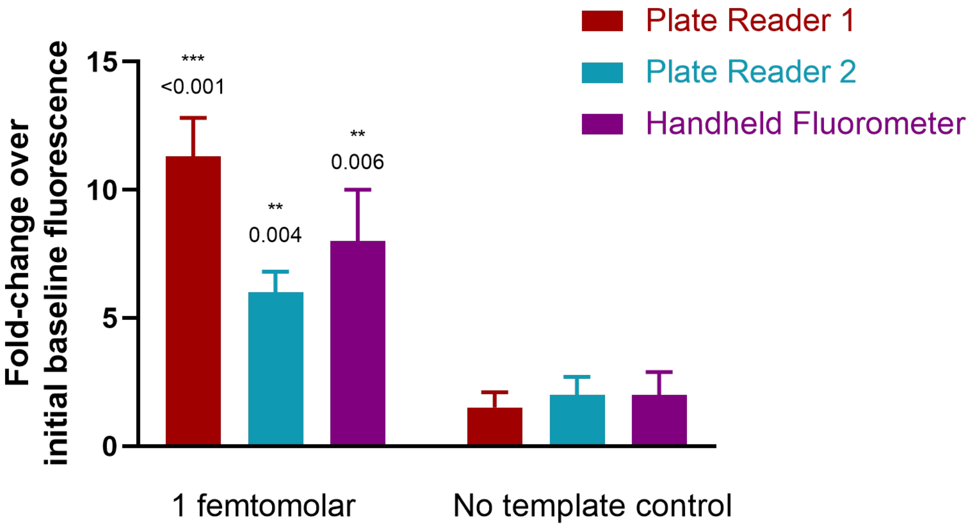

Supp. Fig. 1: Mean fold-change over baseline initial fluorescence of 1 fM (602 parasites/μL) *P. falciparum* whole genomic DNA spiked into whole blood compared with a no-template control in our final assay workflow (10 minutes S-PREP followed by rehydration of *P. falciparum* lyophilized SHERLOCK assay). Asterisks indicate significant differences in fluorescence between simulated sample and no-template control. Error bars indicate standard deviation of three replicates. *p<0.05, **p<0.01, ***<0.001, ****p<0.0001.

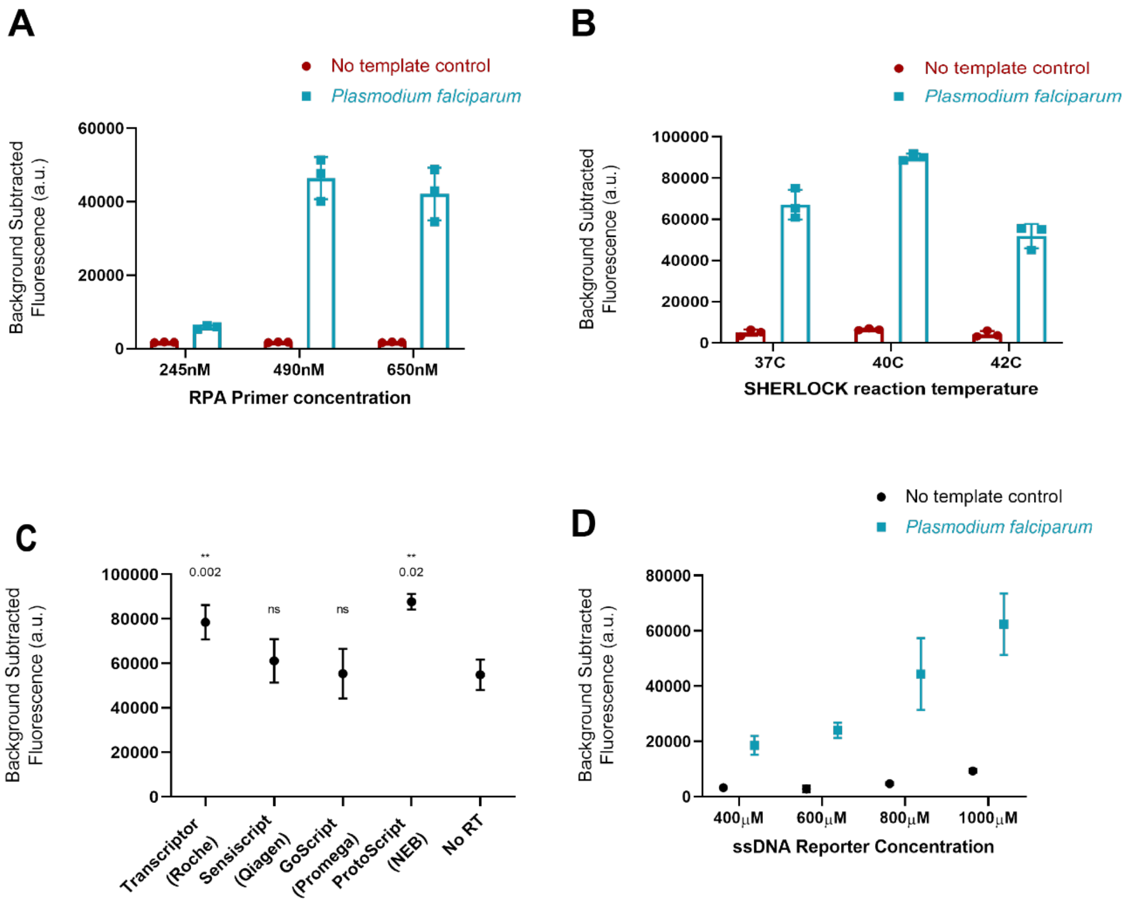

Supp. Fig. 2: Optimization of SHERLOCK parameters, including: A) RPA primer concentration, B) reaction temperature, C) reverse-transcriptase (RT) brand, and D) ssDNA reporter concentration. All optimizations were performed using 1 fM (602 parasites/μL) concentrations of whole genomic *P. falciparum* DNA in water, except for RT brand selection, for which a 1 fM concentration of live intraerythrocytic *P. falciparum* spiked into whole blood was used. Asterisks indicate significant differences in fluorescence of different RT brands from a 1 fM *P. falciparum* reaction where no RT was added assessed by a Student's two-tailed t-test. Bars and lines: mean +/- S.D. of three technical replicates. *p<0.05, **p<0.01, ***<0.001, ****p<0.0001.

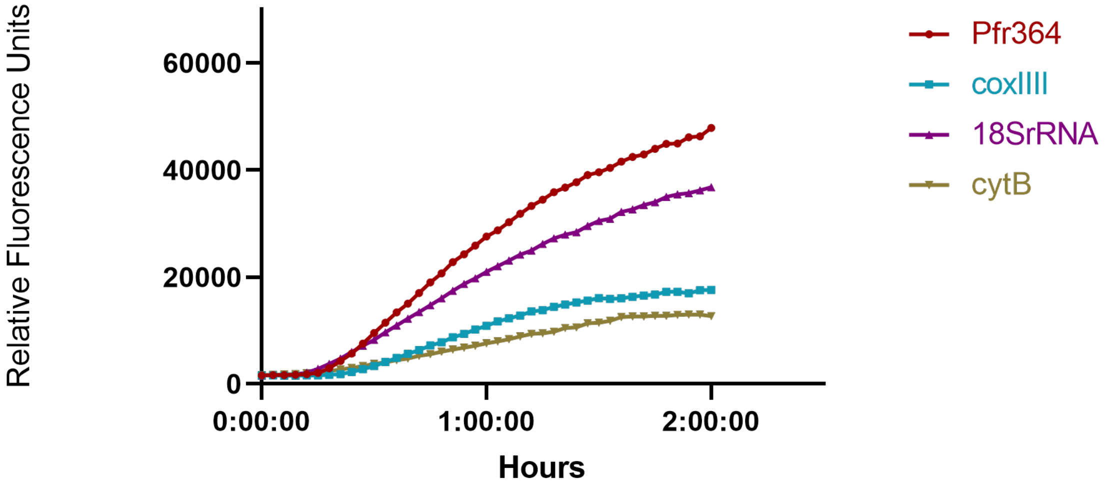

Supp. Fig. 3: Optimization of *P. falciparum* target sequences during RPA screen, comparing best performing primer pairs of each target: Pfr364, cytochrome oxidase III (cox III), 18S rRNA, and cytochrome B (cytB).

	RPA Forward Primer — gRNA match — RPA Reverse Primer
***P. falciparum* sub-telomeric region**	AACGCTGCATTTTGGTCCATTTTTTGGACATTACG-----------------GGTTTCTACGCTTATGTTTT----------------------------GGGCAGGGAAAACATGGGGCAATTGTTCCTTTA
P.vivax LT635620	ATGTGTTATTTTTGGTCCATTTTTTGTCCATTTTT------------------------GCGCCTATTTTTC----------------------------CATTTCTTACGTGTTCGCACAATTCGCCATTTG
P. ovale LT594505.1	TCTTTTTCAACGACATGTCTTTCGTGAATGTTACG--------------------AAATAGAAGGGATAG----------------------------------AGGGAGAACATGGGGCAATTTGTTGTGAC
P.malariae LT594635.1	TTTTTTTACATTTGTTCCATTTTTTGCACATTTTA-----------------GGGTGCTACAAATCCGTTGC------------------------------------ATAACACCAGTTTTGCACGAATTTT

	RPA Forward Primer — gRNA match — RPA Reverse Primer
***P. vivax* mitochondrial region**	CCTTACGTACTCTAGCTTTTAACACAATATTATTGTC-------------TATATTCATTATTCTGAATA---------------------------ACCATATAATTTCAACAAAATGCCAGTATAATATTGT
P.falciparum M76611	CCTTACGTACTCTAGCTATGAACACAATTGTCTATTC-------------CATGTTCATTTATTCTGAATA--------------------------ACCAGATTATTTCAACAAAATGCCAATATAAAATTGT
P. ovale HQ712052	CCTTACGTACTCTAGCTATTTACACAAATATATTATT-------------ATGTTACATTATTCAGAATA---------------------------ACCATATTATTTCTATAAAATGCCAATATAATATTGT
P. malariae LT594637.1	CCTTACGTACTCTAGCTTTGTACACAAATTAATTCGT-------------TATATGTTCAATTATTCTGAA--------------------------ACCATACTATTTCTATAAAATGCCAATATAATATTGT

	RPA Forward Primer — gRNA match — RPA Reverse Primer
***P. ovale* 18S rRNA gene**	AAGTTAAGGGAGTGAAGACGATCAGATACCGTCG----------------AATAAGAAAATTCCTTTCGG-------------------CCTTATGAGAAATCAAAGTCTTTGGGTTCTGGGGCGAGTA
P.falciparum 18S rRNA MN852864.1	AAGTTAAGGGAGTGAAGACGATCAGATACCGTCG----------------AAAAATAAAAGTCATCTTTC-------------------CCTTATGAGAAATCAAAGTCTTTGGGTTCTGGGGCGAGTA
P. vivax DQ660817	AAGTTAAGGGAGTGAAGACGATCAGATACCGTCG----------------AATAAGGATAGTCTCTTCGG-------------------CCCTATGAGAAATCAAAGTCTTTGGGTTCTGGGGCGAGTA
P. malariae AF145336	AAGTTAAGGGAGTGAAGACGATCAGATACCGTCG----------------TAAAAGAGACATTCATATAT-------------------CCTTATGAGAAATCAAAGTCTTTGGGTTCTGGGGCGAGTA

	RPA Forward Primer — gRNA match — RPA Reverse Primer
***P. malariae* 18S rRNA gene**	AACGAAAGTTAAGGGAGTGAAGACGATCAGATACCG------------TTTTAGATAGCTTCCTTCAG---------------------CCTTATGAGAAATCAAAGTCTTTGGGTTCTGGGGCGAGTA
P.falciparum 18S rRNA MN852864.1	AACGAAAGTTAAGGGAGTGAAGACGATCAGATACCG------------TTTCAGATTGCTTCCCTCAG---------------------CCTTATGAGAAATCAAAGTCTTTGGGTTCTGGGGCGAGTA
P. vivax DQ660817	AACGAAAGTTAAGGGAGTGAAGACGATCAGATACCG------------CCTTAGATTTCTTCCTTCAG---------------------CCTTATGAGAAATCAAAGTCTTTGGGTTCTGGGGCGAGTA
P. ovale AB182489.1	AACGAAAGTTAAGGGAGTGAAGACGATCAGATACCG------------TCTTAGATTGCTTCCTTCAG---------------------CCTTATGAGAAATCAAAGTCTTTGGGTTCTGGGGCGAGTA

Supp. Fig. 4: Best performing RPA primers and gRNA sequences for development of *Plasmodium* SHERLOCK assays aligned with the closest homologous genes of other *Plasmodium* species or sequences with lowest matching E-values in NCBI database. First bolded line of each block demonstrates RPA primer and gRNA locations of target sequence in the 5' to 3' direction on the same strand. Alignments below are listed with GenBank accession number; blue color denotes nucleotide matches of RPA forward and reverse primers as well as gRNA sequences.

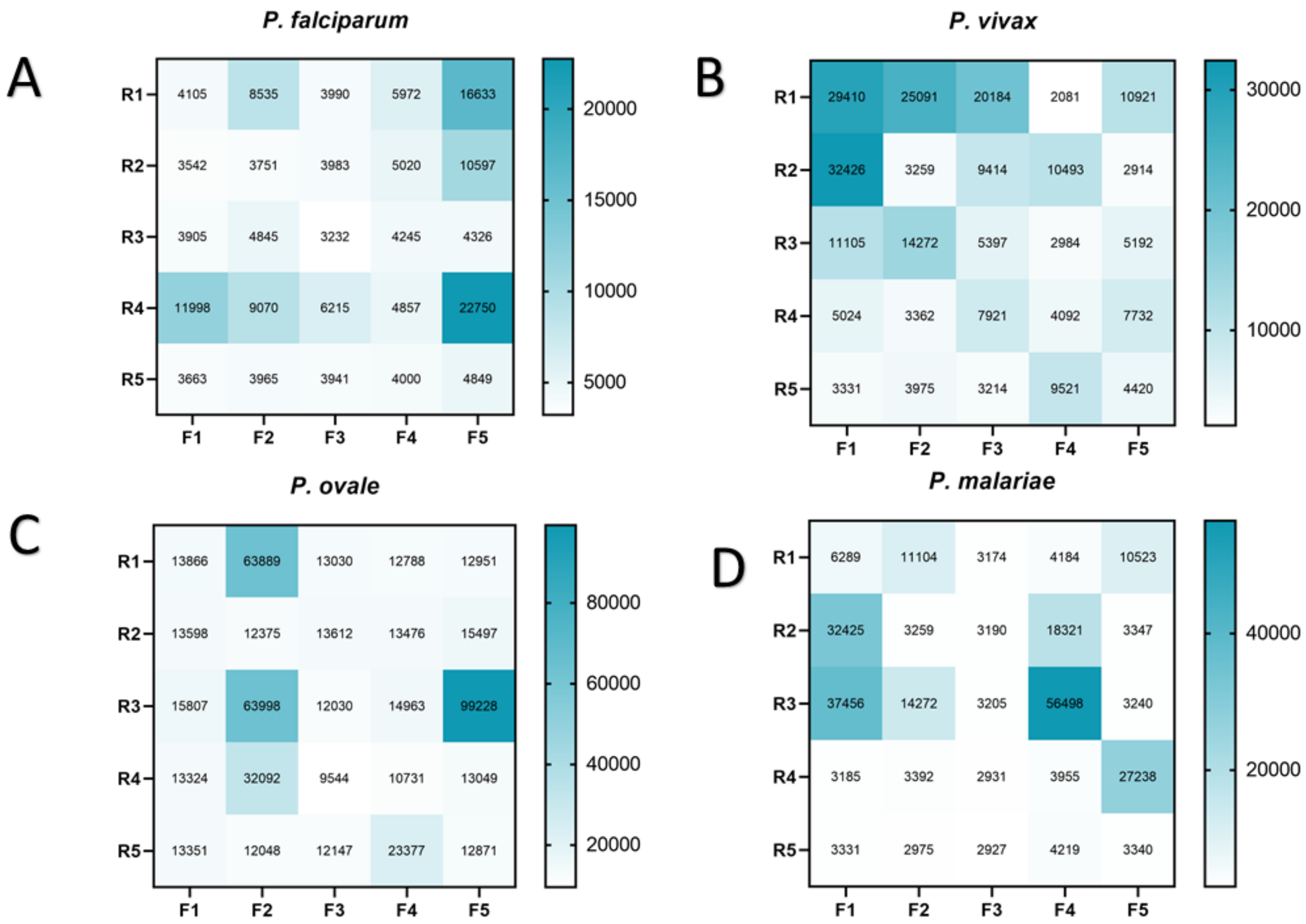

Supp. Fig. 5: Relative fluorescence unit (RFU) measurements of RPA screens comprised of 25-set combination of five forward (F1-F5) and five reverse primers (R1-5) for best-performing sequence targets for *Plasmodium* species targets.

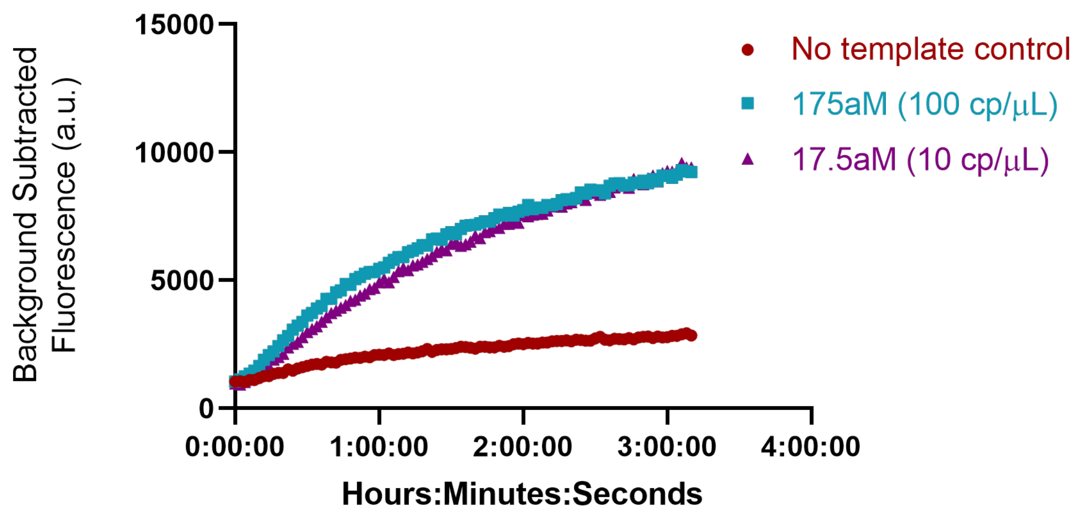

Supp. Fig. 6: Detection of simulated RNA sample (synthetic SARS-CoV-2 synthetic RNA spiked in water) prepared with S-PREP and tested in SARS-CoV-2 SHERLOCK assay

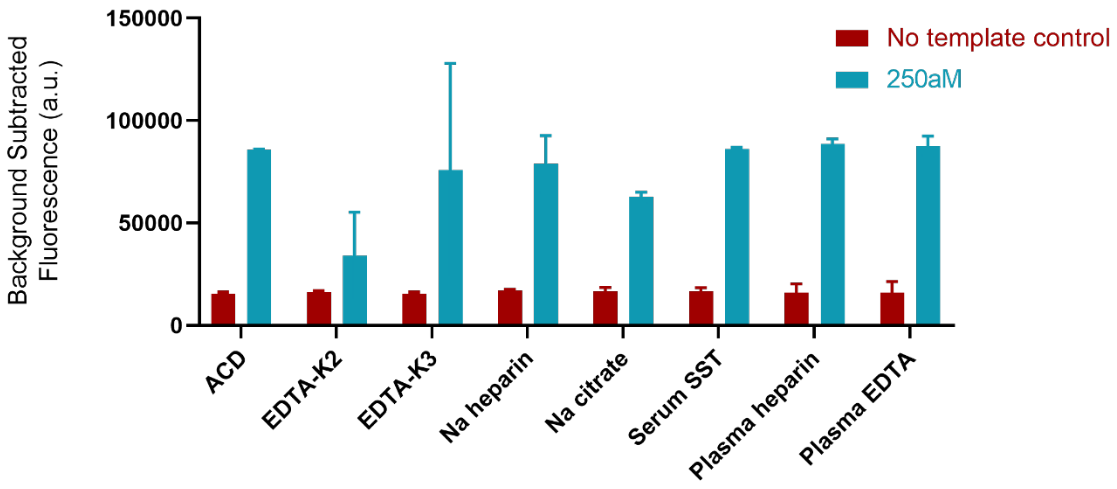

Supp. Fig. 7: Specimen collection types compatible with SHERLOCK tested by spiking live intraerythrocytic *P. falciparum* into whole blood collected in various sample collection tubes to a final concentration of 250 aM (150 parasites/µL) and then prepared with S-PREP (1:3 dilution) for rehydration of lyophilized *P. falciparum* SHERLOCK reaction.

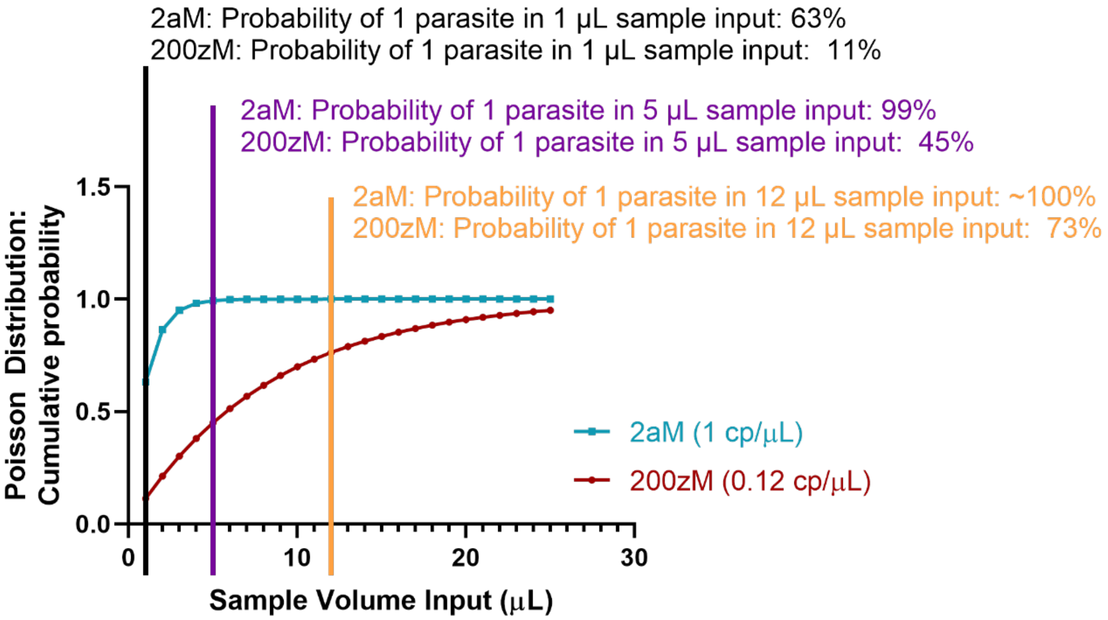

Supp. Fig. 8: Theoretical limits of detection in a perfect assay (one pathogen copy per reaction will be detected 100% of the time) where at attomolar and zeptomolar concentrations of pathogen, a Gaussian distribution is no longer an appropriate approximation of pathogen distribution.
